# Anti-inflammatory, analgesic, and anti-immune-mediated polyarthritis activities of topically applied polarity fractions of *Clematis florida* var. *Plena* and their underlying mechanisms

**DOI:** 10.3389/fvets.2026.1846520

**Published:** 2026-08-26

**Authors:** Ting Lei, Jiabing Zheng, Jie Han, Nian Chen, Jizhou Zhang, Chunquan Zhou, Jie Zhang, Kan Lin, Gong Wang, Li Zhao, Jing Han

**Affiliations:** 1 Institute of Materia Medica, Fujian Academy of Chinese Medical Sciences, Fuzhou, China; 2 Fujian Medical Universitity Union Hospital, Fujian Medical Universtity, Fuzhou, China

**Keywords:** anti-inflammatory activity, *Clematis florida* var. *Plena*, ERK signaling, immune-mediated polyarthritis, NF-κB signaling, polarity-based fractions

## Abstract

**Background:**

*Clematis florida* var. *Plena* (*C. florida*) holds significant potential as a topical treatment for immune-mediated polyarthritis (IMPA); however, its active fractions and mechanisms of action remain poorly understood. Given the chemical complexity of herbal remedies, this study adopted a bioactivity- and mechanism-driven approach, focusing on the effects of polarity-based fractions rather than isolated compounds.

**Methods:**

*C. florida* was extracted with 70% ethanol and sequentially partitioned into petroleum ether (PE), ethyl acetate (EA), *n*-butanol (BU), and water (WA) fractions. Phytochemical analysis was conducted to determine the distribution of saponins and flavonoids. Anti-inflammatory and analgesic activities were evaluated using xylene-induced ear edema and acetic acid-induced writhing models, respectively. Therapeutic efficacy was assessed in a rat model of adjuvant-induced arthritis via topical application. Joint tissues were analyzed using H&E and Safranin O–Fast Green staining, immunohistochemistry, and Western blotting to investigate histological changes and signaling pathways (ERK and NF-κB).

**Results:**

Phytochemical analysis revealed that the extract is rich in saponins and flavonoids, which exhibit distinct distribution patterns across fractions of varying polarity. All fractions exhibited anti-inflammatory effects in the ear edema model and analgesic activity in the writhing test. In the arthritis model, topical application of the EA, BU, and WA fractions significantly attenuated paw swelling and reduced serum levels of TNF-α, COX-2, rheumatoid factor, and IL-6. Histological staining confirmed partial protection against joint destruction. Mechanistic studies revealed that these fractions enhanced ERK phosphorylation and suppressed NF-κB activation in joint tissues.

**Conclusion:**

The EA, BU, and WA fractions of *C. florida* possess significant topical therapeutic potential against IMPA. The anti-arthritic effects are likely associated with the modulation of ERK and NF-κB signaling pathways. This study elucidates the pharmacodynamic basis of *C. florida* fractions, positioning them as promising candidates for the development of topical treatments for animal joint disorders.

## 1 Introduction

Immune-mediated polyarthritis (IMPA) is a major cause of joint pain and locomotor dysfunction in canine and feline clinical practice (1, 2). It is fundamentally characterized by an aberrant immune response directed against articular tissues (3, 4). Current therapeutic strategies rely heavily on glucocorticoids and conventional immunosuppressive agents. Although effective, the long-term administration of these drugs is often associated with significant toxicity and adverse effects, posing a major clinical challenge (5–8). Rheumatoid arthritis (RA), the prototypical autoimmune arthritis, exhibits strong pathogenic overlap with veterinary IMPA (9, 10). Consequently, the adjuvant-induced arthritis (AA) rat model provides a valuable tool for evaluating novel therapeutics, offering translational relevance for both human and animal health.

Traditional Chinese Medicine (TCM) presents a promising therapeutic approach for IMPA management, attributed to its inherent characteristics of multi-component synergy, multi-target regulation, and low toxicity (11). In recent years, numerous clinical trials and experimental studies have systematically validated the pharmacological activities and underlying mechanisms of multiple bioactive components derived from TCM, underscoring their significant application value and development potential in IMPA treatment (12), thereby providing abundant theoretical and material support for the development of innovative anti-IMPA drugs.

*Clematis florida* var. *Plena* (*C. florida*), a member of the genus *Clematis* in the Ranunculaceae family, is a well-documented traditional herbal medicine in southeastern China, traditionally utilized for the treatment of rheumatic pain (13, 14). As recorded in *Quan Guo Zhong Cao Yao Hui Bian*, topical application of *C. florida* exhibits promising applicability in alleviating joint swelling and pain (15). In traditional folk medicine, the herb *C. florida* has long been used either orally or topically, prepared either as an ethanol extract or formulated in camellia seed oil as a base, for the management of traumatic injuries, arthritis, and other inflammatory conditions.

Our previous work has revealed that *C. florida* contains a diverse array of bioactive components, including alkaloids, flavonoids, fatty acids, and triterpenoid saponins (16). Other pharmacological studies further confirm that *C. florida* extracts, particularly those enriched with triterpenoid saponins and alkaloids, exert significant anti-tumor and anti-inflammatory effects (17–19). Despite these findings, pharmacological research on *C. florida* remains relatively limited, with most studies focusing exclusively on its anti-inflammatory properties rather than exploring its broad efficacy, especially in arthritis treatment.

The pharmacological properties of botanical extracts are closely related to their chemical composition, which is strongly influenced by the polarity of the extraction solvent (20). Polarity-based fractionation can yield extracts enriched in distinct classes of secondary metabolites, potentially resulting in differential biological activities (21). Although *C. florida* has been reported to exhibit anti-inflammatory effects, systematic pharmacological evaluations of polarity-defined *C. florida* extracts—particularly following topical administration—remain limited. Clarifying the relationship between extract polarity and pharmacological efficacy is therefore essential for advancing the rational development of plant-derived topical agents for inflammatory diseases such as IMPA.

To systematically investigate polarity-dependent pharmacological effects, liquid–liquid extraction is widely employed in pharmaceutical research to fractionate crude botanical extracts according to solvent polarity (22–24). In our previous study, we combined LC–MS/MS–based phytochemical profiling with network pharmacology analysis and identified extracellular signal–regulated kinase (ERK)/mitogen-activated protein kinase (MAPK) and nuclear factor–kappa B (NF-κB) as key signaling pathways potentially mediating the anti-IMPA effects of the ethanol extract of *C. florida*. These predictions were subsequently supported by *in vivo* experimental validation using an AA rat model (16). However, given that both the composition and relative abundance of bioactive constituents can differ substantially among polarity-based fractions, it remains unclear whether individual fractions of *C. florida* retain comparable anti-IMPA efficacy or engage similar molecular pathways when applied topically.

**Figure 1:**
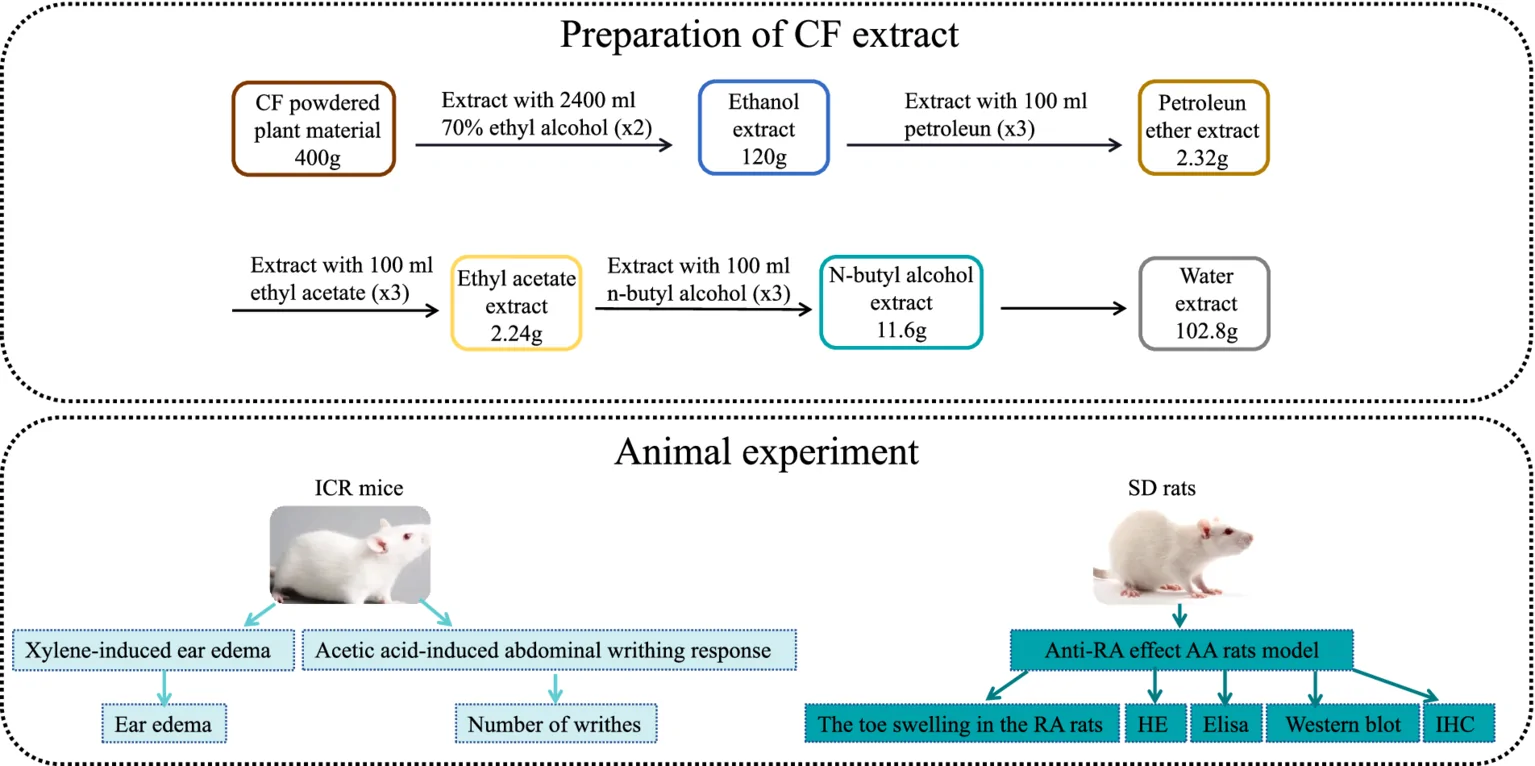
Schematic overview of the experimental workflow evaluating the anti-inflammatory, analgesic, and anti-IMPA activities of *C. florida* extracts with varying polarities.

Accordingly, the present study aimed to systematically evaluate the anti-inflammatory, analgesic, and anti-IMPA potential of four polarity-defined *C. florida* extracts using a panel of established animal models. Serum levels of key inflammatory mediators were quantified by enzyme-linked immunosorbent assay (ELISA), while immunohistochemistry and Western blot analyses were employed to assess the involvement of ERK- and NF-κB–related signaling pathways in the ankle joint tissues of AA rats. A detailed overview of the research workflow, spanning from *C. florida* extract preparation to the execution of *in vivo* animal experiments, is illustrated in Figure 1.

## 2 Materials and methods

### 2.1 Plant collection and identification

*C. florida*, authenticated by Professor Zehao Huang of Fujian University of Traditional Chinese Medicine (25), was purchased from Fuan Shecaotang Ecological Agriculture Co., Ltd. (Ningde, China, 20240128). The taxonomic and morphological identification details of *C. florida* are provided in Table 1.

**Table 1:** Taxonomic and morphological identification details of *C. florida*.

Items	Description
Date of collection	September 25, 2023
Collection number	20230925
Family name	Ranunculaceae
Generic name	*Clematis*
Scientific name	*Clematis florida* var. *Plena*
Periderm	Poorly developed, no periderm present
Epidermis	Single layer of cells with thickened outer walls, brownish-yellow in color
Cortex	Relatively wide, composed of over 10 layers of nearly circular parenchyma cells containing abundant starch grains
Endodermis	Distinct; Casparian dots clearly visible in cross-section
Vascular bundle type	Collateral
Primary xylem	Diarch (two protoxylem poles), with two distinct concave arcs
Phloem	More developed on the outer sides of the two concave arcs of the xylem
Xylem cells	Fully lignified, vessels with large lumens, interfascicular cells often fibrous, with thick walls, distinct lamellae, and extremely narrow lumens
Powder–Fibers	Fusiform or elongated, yellowish-brown, 14–27 μm in diameter, 100–199 μm long, wall thickness 3–8 μm, lignified, with dense pits and frequent pit canals
Powder–Other features	Includes stone cells, fiber tracheids, bordered-pitted/netted/spiral vessels, brownish to reddish-brown amorphous masses, and numerous starch grains
Habitat	Subtropical and tropical regions of southeastern China

### 2.2 Preparation of *C. florida* extract

Dried plant material (400 g) was ground into a coarse powder and subjected to heat-reflux extraction with 70% aqueous ethanol (v/v) in a round-bottom flask at 90 °C for 2 h. After cooling to room temperature, the mixture was filtered, and the residual plant material was re-extracted twice under identical conditions. The combined filtrates were concentrated under reduced pressure using a rotary evaporator to remove ethanol and excess water, yielding a crude ethanolic extract of *C. florida* (ET, 120 g).

This crude extract was suspended in deionized water and sequentially partitioned with petroleum ether, ethyl acetate, and n-butanol. Following each extraction step, the organic layers were separated, and the remaining aqueous phase was used for the subsequent partition. All fractions—including the petroleum ether, ethyl acetate, n-butanol, and final aqueous layers—were concentrated under vacuum to dryness, affording the following yields: petroleum ether fraction (PE, 2.32 g), ethyl acetate fraction (EA, 2.24 g), *n*-butanol fraction (BU, 11.6 g), and water fraction (WA, 102.8 g).

Each fraction and the crude extract were then suspended in an appropriate volume of distilled water to prepare high-dose administration solutions with concentrations of 3.2 g/mL (ET), 0.06 g/mL (PE and EA fractions), 0.3 g/mL (BU), and 2.7 g/mL (WA). The resulting suspensions were stored at 4 °C and freshly diluted with deionized water immediately prior to administration. Each milliliter of the aforementioned extract is equivalent to 10.8 g of raw herbal material.

### 2.3 Phytochemical analysis

The chemical constituents of the extracts were analyzed using a liquid chromatography coupled with high-resolution mass spectrometry (LC–HRMS) system. Chromatographic separation was performed on a Waters Acquity UPLC system equipped with a Kinetex C18 column (2.6 μm, 100 mm × 2.1 mm). The mobile phase consisted of acetonitrile (A) and water containing 0.1% formic acid (B) at a flow rate of 0.4 mL/min. The column temperature was maintained at 30 °C, and the injection volume was 10 μL. The gradient elution program was as follows: 0–1.5 min, 5% A; 1.5–3.5 min, 5–10% A; 3.5–16.5 min, 10–20% A; 16.5–23.5 min, 20–35% A; 23.5–30.5 min, 35–55% A; 30.5–38.5 min, 55–95% A; 38.5–43.0 min, 95% A; followed by a rapid return to 5% A in 0.5 min and re-equilibration for 1.5 min. Mass spectrometric detection was carried out on a Thermo Scientific Q Exactive HF high-resolution mass spectrometer equipped with a heated electrospray ionization (HESI) source. Data acquisition was performed in both positive and negative ion modes over a mass range of *m/z* 100–1,500.

The total flavonoid content was determined using a colorimetric assay (26). Briefly, 3.0 mL of the test sample solution was accurately pipetted into a 25 mL volumetric flask. The volume was adjusted to 6.0 mL with 30% (v/v) ethanol, followed by the addition of 1.0 mL of 5% (w/v) sodium nitrite solution. After thorough mixing, the solution was allowed to stand for 6 min. Subsequently, 1.0 mL of 10% (w/v) aluminum nitrate solution was added, and the mixture was vortexed and incubated for another 6 min. Next, 10.0 mL of 4% (w/v) sodium hydroxide solution was introduced, and the final volume was diluted to the mark with 30% ethanol. Once fully mixed, the solution was left to stand for 15 min at room temperature. Absorbance was measured at 510 nm against a reagent blank prepared under identical conditions but without the sample.

The total saponin content was assessed using the vanillin–glacial acetic acid–perchloric acid colorimetric method (27). An accurately measured volume of the test sample solution was transferred to a test tube and evaporated to dryness in a water bath maintained at 90–100 °C. To the dried residue, 0.2 mL of 5% (w/v) vanillin in glacial acetic acid and 0.8 mL of concentrated perchloric acid were sequentially added. The mixture was gently vortexed and then heated in a water bath at 60 °C for 20 min. The reaction was terminated by placing the tube in an ice bath for 10 min. Thereafter, 5.0 mL of glacial acetic acid was added, and the mixture was homogenized and allowed to stand for 10 min at room temperature. Absorbance was measured at 550 nm against a blank control prepared in parallel, omitting the sample.

### 2.4 Animal models and drug administration

A total of 256 specific pathogen-free (SPF) male ICR mice (18–22 g) and 104 Sprague–Dawley (SD) rats (52 males and 52 females, 170–230 g) were obtained from Beijing Huafukang Bioscience Co., Ltd. (Beijing, China). All animals were housed under standard laboratory conditions (12-h light/dark cycle, 22 ± 2 °C, 50% ± 10% humidity) with ad libitum access to a standardized commercial rodent diet and autoclaved drinking water. All experimental procedures involving animals were reviewed and approved by the Institutional Animal Care and Use Committee of Fujian University of Traditional Chinese Medicine (Approval No.: FJATCM-IACUC2023024).

#### 2.4.1 Xylene-induced mouse ear edema

A total of 128 male ICR mice were randomly allocated using a computer-generated random number sequence to 16 experimental groups: a positive control group treated with diclofenac diethylamine (DD, Novartis Pharma, Beijing, China), a vehicle control group receiving 0.9% saline, high- and low-dose ethanol extract groups (ET-H and ET-L: 0.8 and 0.4 g/mL, respectively), petroleum ether fraction groups (PE-H, PE-M, PE-L: 0.06, 0.03, and 0.015 g/mL), ethyl acetate fraction groups (EA-H, EA-M, EA-L: 0.06, 0.03, and 0.015 g/mL), *n*-butanol fraction groups (BU-H, BU-M, BU-L: 0.3, 0.15, and 0.075 g/mL), and aqueous fraction groups (WA-H, WA-M, WA-L: 2.7, 1.35, and 0.68 g/mL). The group size (*n* = 8) was selected based on commonly used sample sizes in comparable anti-inflammatory studies (28), which have demonstrated sufficient sensitivity to detect treatment effects while adhering to the 3R principles of animal research. The concentrations of the herbal formulations were selected based on preliminary *in vivo* experiments, which identified the lowest doses showing significant anti-inflammatory effects without skin irritation. These doses were neither standardized to specific active compounds nor matched to diclofenac in terms of pharmacokinetic exposure.

A volume of 20 μL of the respective test solution was topically applied to both the inner and outer surfaces of the right ear. For the DD group, a thin layer (approximately 1 mm thick) of diclofenac diethylamine gel was applied. Treatments were administered at 10-min intervals for a total of six applications. Ten minutes after the final dose, 40 μL of xylene was applied to the right ear to induce inflammation, while the left ear remained untreated as an internal control. Thirty minutes later, the mice were euthanized by cervical dislocation, and ear biopsies (6 mm in diameter) were obtained using a sterile biopsy punch. The weight difference between the right (xylene-treated) and left (untreated) ear punches was used to quantify ear edema.

#### 2.4.2 Acetic acid-induced abdominal writhing response

A total of 128 male ICR mice were randomly assigned to 16 experimental groups according to the grouping criteria outlined in Section 2.4.1. One day prior to the experiment, the abdominal fur of each mouse was removed using an 8% sodium sulfide solution. Two days after depilation, mice in the drug-treated groups received a topical application of 0.05 mL of *C. florida* extract to the shaved abdominal area. Mice in the vehicle control group received an equivalent volume of 0.9% saline, while those in the positive control group were treated with 0.05 g of DD. All treatments were administered once daily for five consecutive days. On day 5, the animals received two applications spaced 30 min apart. One hour after the final dose, each mouse was intraperitoneally injected with 0.2 mL of 0.6% acetic acid to induce nociceptive writhing. A writhe was defined as a stereotypical response comprising abdominal constriction, pelvic rotation, and extension of the hind limbs. The number of writhes per animal was recorded during the 5–20-min interval following acetic acid injection.

#### 2.4.3 Adjuvant-induced arthritis (AA) model in rats

A total of 104 Sprague–Dawley (SD) rats were randomly assigned using a computer-generated random number sequence to 13 experimental groups (*n* = 8 per group): a normal control group (0.9% saline), a model control group (0.9% saline), a positive drug group treated with DD, high- and low-dose ethanol extract groups (ET-H and ET-L: 0.8 and 0.4 g/mL), petroleum ether fraction groups (PE-H and PE-L: 0.06 and 0.03 g/mL), ethyl acetate fraction groups (EA-H and EA-L: 0.06 and 0.03 g/mL), *n*-butanol fraction groups (BU-H and BU-L: 0.3 and 0.15 g/mL), and aqueous fraction groups (WA-H and WA-L: 2.7 and 1.35 g/mL). The group size (*n* = 8) was selected based on commonly used sample sizes in comparable anti-arthritic studies (29).

Rats were gently restrained in a supine position using a dedicated immobilizer. Complete Freund’s adjuvant (CFA; 0.1 mL, Beyotime, Shanghai, China) was injected intradermally into the plantar surface of the left hind paw. One minute after injection, the animals were returned to their home cages. All groups except the normal control group received CFA to induce adjuvant-induced arthritis (AA). The AA model serves as a surrogate for the inflammatory and immune-driven features of RA and IMPA, but does not replicate the full clinical complexity of these diseases.

Starting on day 2 post-CFA injection, the drug-treated groups received daily topical applications of the corresponding *C. florida* extract to the inflamed paw for 28 consecutive days. The positive control group received a daily topical dose of 0.2 g diclofenac diethylamine gel (equivalent to a diclofenac dose of 10 mg/kg body weight). The model and normal control groups received an equivalent amount of 0.9% saline applied to the left hind paw.

Body weight and paw volume were measured on the day before CFA injection and on days 7, 14, 21, and 28 after CFA injection. Paw volume was assessed using a plethysmometer (KEW, Shanghai, China); the average of five consecutive measurements was recorded for each paw. Paw swelling (%) was calculated using the following formula: [(*V_t_* − *V_0_*)/*V_0_*] × 100%, where *V_t_* represents the paw volume at time *t*, and *V_0_* is baseline paw volume prior to CFA injection.

Arthritis severity was evaluated on days 1, 7, 14, 21, and 28 post-CFA injection using a standardized scoring system: 0 = no visible joint inflammation; 1 = erythema and mild swelling confined to the small toe joints; 2 = erythema and swelling involving the toes and metatarsal joints; 3 = inflammation extending to the ankle joint; and 4 = severe erythema and swelling affecting the entire paw and extending to the ankle. Each limb was scored individually, and the total arthritis index per rat was calculated as the sum of scores from all four limbs, with a maximum possible score of 16. Arthritis severity was scored by two independent observers blinded to the experimental groups, using a semi-quantitative scoring system.

After 28 days of treatment, the rats were euthanized under deep anesthesia induced by an intraperitoneal injection of sodium pentobarbital. Blood was collected via cardiac puncture, and the spleens were aseptically harvested. The spleens were immediately rinsed in ice-cold phosphate-buffered saline, gently blotted dry with sterile filter paper, and weighed. The spleen index was calculated as the ratio of spleen weight to the body weight of the rat. Additionally, ankle joints and synovial tissues from the knee joints were dissected and collected for subsequent histopathological and molecular analyses. Whole blood samples were centrifuged at 3000 × g for 15 min at 4 °C to isolate serum, which was aliquoted and stored at −80 °C until further use.

### 2.5 Hematoxylin and eosin staining

Ankle joint tissues were harvested and immediately fixed in 4% paraformaldehyde. Following fixation, the samples were decalcified in 10% EDTA (pH 7.4), with the decalcifying solution changed every 3 days until decalcification was complete, as confirmed by the lack of resistance to needle probing. The decalcified tissues were then dehydrated through a graded ethanol series, cleared in xylene, and embedded in paraffin. Serial sagittal sections were cut using a microtome and mounted on glass slides.

For hematoxylin and eosin (H&E) staining, the sections were deparaffinized in xylene, rehydrated through a descending ethanol series, and stained with Harris hematoxylin for 5 min. Nuclei were differentiated in 1% acid alcohol for 30 s, followed by bluing in tap water. The sections were then counterstained with 0.5% eosin Y for 10 min, dehydrated through an ascending ethanol series, cleared in xylene, and coverslipped with a permanent mounting medium. Histopathological evaluation was performed under a light microscope (Leica Microsystems, Germany) at 200 × magnification.

Histopathological changes were evaluated using a semi-quantitative scoring system by two independent observers blinded to the experimental groups. The severity of synovial inflammation, cartilage damage, and bone destruction was graded on a scale of 0 to 3 as follows: 0 = normal; 1 = mild (slight synovial hyperplasia or focal inflammatory cell infiltration); 2 = moderate (moderate synovial thickening, multifocal inflammatory infiltration, or mild cartilage erosion); and 3 = severe (marked synovial hyperplasia, dense inflammatory infiltration, pannus formation, or severe cartilage and bone destruction).

### 2.6 Safranin O–fast green staining

Ankle joint tissues were harvested, fixed in 4% paraformaldehyde, decalcified in 10% neutral buffered EDTA, embedded in paraffin, and cut into 5-μm-thick sections. Prior to staining, the sections were deparaffinized in xylene and rehydrated through a descending ethanol series to distilled water.

For Safranin O–Fast Green staining, the sections were first immersed in 0.1% Fast Green solution for 3 min, followed by brief differentiation in 1% acetic acid for 10–15 s to remove excess background stain. After rinsing in distilled water, the sections were stained with 0.1% Safranin O solution for 3 min. The slides were then dehydrated through an ascending ethanol series, cleared in xylene, and coverslipped with a permanent mounting medium. The stained sections were examined under a light microscope (Leica Microsystems, Germany). The scoring criteria were defined as follows: 0 = Normal, characterized by a smooth articular surface and intense, uniform Safranin O staining throughout the full depth of the cartilage; 1 = Mild, showing a smooth surface but with slight depletion of Safranin O staining in the superficial zone; 2 = Moderate, indicating surface fibrillation or superficial clefts accompanied by moderate loss of matrix staining extending to the middle zone; 3 = Severe, defined by deep clefts extending into the deep zone with severe proteoglycan depletion; and 4 = End-stage, representing complete erosion of the cartilage down to the subchondral bone with total loss of Safranin O staining. All sections were graded by two independent observers blinded to the experimental groups.

### 2.7 Determination of serum inflammatory factor

Following the final paw volume measurement, the rats were euthanized under deep anesthesia induced by intraperitoneal administration of sodium pentobarbital. Blood was collected from the abdominal aorta and immediately transferred to serum separator tubes. The samples were allowed to clot at room temperature for 30 min and then centrifuged at 3,000 × g for 10 min at 4 °C to isolate serum. The resulting serum was aliquoted and stored at −80 °C until analysis.

Serum levels of COX-2, TNF-α, IL-6, IL-1β, and RF were quantified using commercial ELISA kits according to the manufacturer’s instructions (Elabscience, Wuhan, China).

### 2.8 Western blotting

Joint tissues were harvested immediately after the final paw volume measurement. Total protein was extracted using a column-based tissue protein extraction kit (Yaenzyme, Shanghai, China). Briefly, 10 mg of tissue was homogenized in 100 μL of ice-cold lysis buffer using a high-speed cryogenic tissue grinder (Servicebio, Wuhan, China). The resulting homogenate was transferred to an ultrafiltration centrifuge tube and centrifuged at 12,000 × g for 2 min at 4 °C. The supernatant was collected, and the protein concentration was determined using a BCA Protein Assay Kit (Yaenzyme, Shanghai, China), with bovine serum albumin (BSA) as the standard.

Equal amounts of protein were separated by sodium dodecyl sulfate–polyacrylamide gel electrophoresis (SDS-PAGE) and subsequently transferred onto a polyvinylidene difluoride (PVDF) membrane. The membranes were blocked with 5% nonfat milk or the manufacturer-recommended blocking solution in Tris-buffered saline containing 0.1% Tween-20 (TBST) to prevent non-specific binding. The membranes were then incubated overnight at 4 °C with the following primary antibodies: p-ERK1/2 (Thr202/Tyr204; Cell Signaling Technology, USA, #3033S, 1:4000), total ERK1/2 (Cell Signaling Technology, USA, #4695S, 1:1000), NF-κB p65 (phospho-Ser536) (BIOSS, China, bsm-33117M, 1:1000), and NF-κB p65 (total) (ABclonal, China, A4782, 1:2000). After three washes with TBST, the membranes were incubated for 2 h at 26 °C with horseradish peroxidase-conjugated secondary antibodies (BIOSS, China, 1:10,000). Following three additional TBST washes, immunoreactive bands were visualized using an enhanced chemiluminescence substrate (Yaenzyme, China), and the band intensities were measured using ImageJ (version 1.53, National Institutes of Health, Bethesda, MD, USA). Data represent the mean of three biological replicates (individual animals).

### 2.9 Immunohistochemistry analysis of tissue sections

Tissue samples were collected, fixed, decalcified, embedded in paraffin, and sectioned at a thickness of 5 μm. Joint sections were deparaffinized in xylene and rehydrated through a graded ethanol series. Antigen retrieval was performed by incubating the sections in citrate buffer (pH 6.0) at 98 °C for 10 min. After the sections were cooled to room temperature for 20 min, endogenous peroxidase activity was quenched by incubation in 3% hydrogen peroxide for 15 min. The sections were then incubated overnight at 4 °C with primary antibodies against p-ERK1/2 (1:200), total ERK1/2 (1:50), NF-κB p65 (phospho-Ser536; 1:400), and NF-κB p65 (total; 1:200). Following three washes with PBS, the sections were incubated with an HRP-conjugated secondary antibody at room temperature for 15 min. Subsequently, color development using 3,3′-diaminobenzidine (DAB), counterstaining with hematoxylin, differentiation, bluing, dehydration, and mounting were carried out according to standard protocols. The stained slides were examined under a light microscope. Positive immunoreactivity for p-ERK1/2, ERK, NF-κB p65, and NF-κB was visualized as brown staining in the tissue.

### 2.10 Statistical analysis

All data are expressed as the mean ± standard error of the mean (SEM). Normality of distribution and homogeneity of variances were assessed using the Shapiro–Wilk test and Levene’s test, respectively. When the assumptions of normality and equal variance were met, differences among groups were evaluated by one-way analysis of variance (ANOVA). If the ANOVA revealed a significant effect, *post hoc* comparisons were conducted using Dunnett’s test to compare each treatment group against the model group. If the assumption of homogeneity of variance was violated, Welch’s ANOVA followed by the Games–Howell *post hoc* test was used instead. Effect sizes for pairwise comparisons were reported as Cohen’s *d*. Statistical significance was defined as *p* < 0.05, and all tests were two-tailed. All analyses were performed using IBM SPSS Statistics software (version 20.0, IBM Corp., Armonk, NY, USA).

## 3 Results

### 3.1 Phytochemical analysis of *C. florida* extracts

Comprehensive chemical profiling of the extracts was performed using LC-HRMS in both positive and negative ionization modes. The total ion chromatograms (TIC) and the detailed list of identified compounds are presented in Supplementary Figure S1 and Supplementary Table S1, respectively. The analysis identified 48 compounds with high confidence, including flavonoids, saponins, lignans, alkaloids, and phenolic acids. These compounds were characterized based on their *m/z* values, retention times, and ion adduct patterns. The results provide a comprehensive phytochemical profile of the extracts, highlighting their complex chemical composition.

The total flavonoid content, expressed as rutin equivalents, varied significantly among the solvent fractions (*p* < 0.001, Cohen’s *f* = 4.776). The WA fraction exhibited the highest total flavonoid content (1.023 ± 0.092 mg/mL), followed by the EA (0.710 ± 0.046 mg/mL), BU (0.581 ± 0.034 mg/mL), and PE fractions (0.294 ± 0.018 mg/mL).

Similarly, total saponin content, quantified as oleanolic acid equivalents, showed a pronounced solvent-dependent distribution (*p* < 0.001, Cohen’s *f* = 16.515). The WA layer contained the highest saponin level (31.703 ± 1.454 mg/mL), markedly exceeding those of the BU (6.267 ± 0.247 mg/mL), EA (4.868 ± 0.187 mg/mL), and PE (2.498 ± 0.068 mg/mL) fractions. This suggests that flavonoids and saponins are predominantly enriched in the more polar solvent fractions.

### 3.2 Anti-inflammatory effects of *C. florida* extracts on xylene-induced ear edema

**Figure 2:**
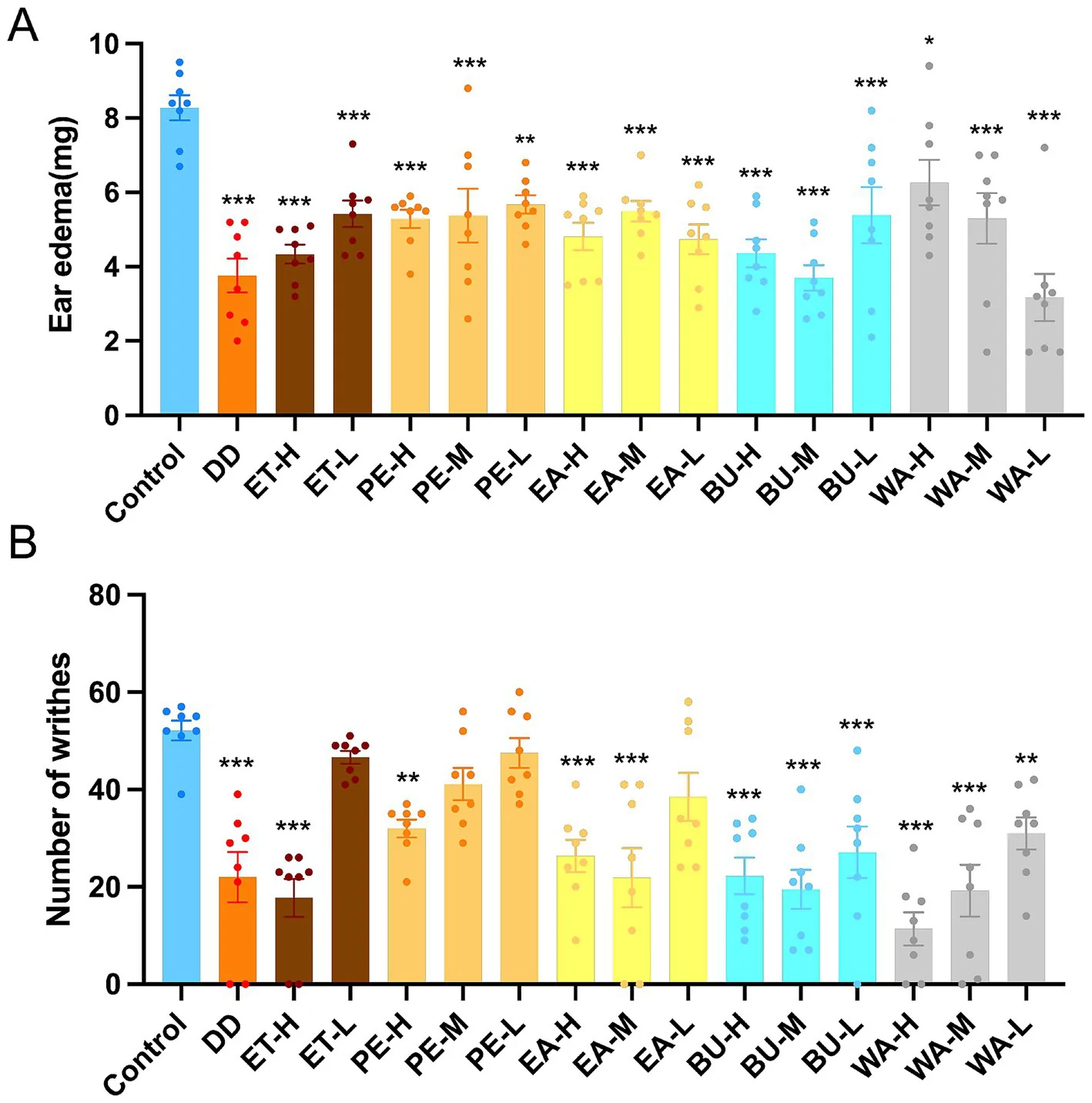
Anti-inflammatory and analgesic effects of *C. florida* extracts with different polarities (*n* = 8 per group). **(A)** Inhibitory effects of different polarities on ear edema in mice. **(B)** Inhibitory effects of different polarities on writhing frequency in mice. Data are presented as mean ± SEM. Statistical comparisons were performed using one-way ANOVA followed by Dunnett’s *post hoc* test. ^**^p < 0.01, ^***^p < 0.001 vs. control group.

The results of the xylene-induced ear edema assay are presented in Figure 2A. Compared with the vehicle control group, the various *C. florida* extracts and fractions significantly attenuated ear edema, indicating potent anti-inflammatory activity (all *p* < 0.05; see Supplementary Table S2). Notably, the BU-M and WA-L groups exhibited the most pronounced inhibitory effects (*p* < 0.0001). Moreover, the ET, PE, and BU treatment groups demonstrated a reduction in edema formation in a dose-dependent manner.

### 3.3 Effects of *C. florida* extracts on acetic acid-induced writhing response

As shown in Figure 2B, administration of extracts derived from various fractions of *C. florida* significantly attenuated the acetic acid–induced writhing response compared to the vehicle control group, albeit with varying efficacy. Among them, the ET-H, BU-M, and WA-H groups demonstrated the most pronounced analgesic effects (all *p* < 0.001; see Supplementary Table S3 for exact *p* values and effect sizes). Furthermore, a clear dose-dependent analgesic trend was observed for both the PE and WA fractions, with high-dose treatments yielding statistically significant reductions in writhing frequency.

### 3.4 Effects of *C. florida* fractions on AA rats

#### 3.4.1 Effects of *C. florida* fractions on paw swelling rate, arthritis score, spleen index, and body weight in AA rats

**Figure 3:**
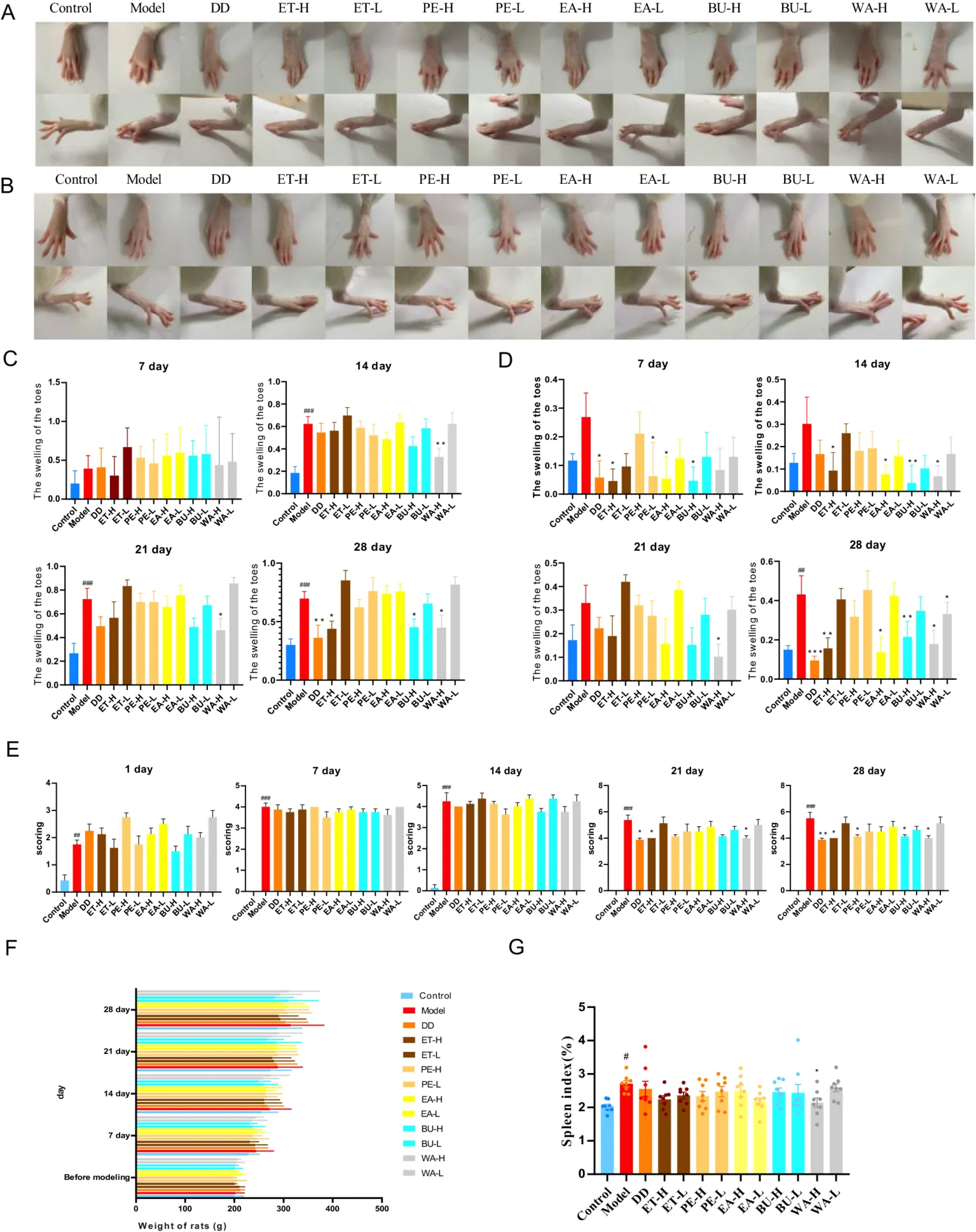
Effects of *C. florida* fractions on AA rats (*n* = 8 per group). **(A)** Representative images of left hind paw swelling in each group of rats. **(B)** Representative images of right hind paw swelling in each group of rats. **(C)** Effects of *C. florida* fractions on left hind paw swelling in AA rats. **(D)** Effects of *C. florida* fractions on right hind paw swelling in AA rats. **(E)** Effects of *C. florida* fractions on arthritis scores in AA rats. **(F)** Effects of *C. florida* fractions on the body weight of AA rats. **(G)** Effects of *C. florida* fractions on spleen index in AA rats. Data are presented as mean ± SEM. Statistical comparisons were performed using one-way ANOVA followed by Dunnett’s *post hoc* test. ^#^*p* < 0.05, ^##^*p* < 0.01, ^###^*p* < 0.001 vs. Control group; ^*^p < 0.05, ^**^p < 0.01, ^***^p < 0.001 vs. model group.

The ET-H, BU-H, and WA-H fractions of *C. florida* demonstrated significant therapeutic effects in AA rats, with responses exhibiting dose-dependent trends. As shown in Figures 3A–D, 14 days after CFA injection, the WA-H group exhibited a marked reduction in primary paw swelling (*p* = 0.018, Cohen’s *d* = 1.346). In contrast, the ET-H (*p* = 0.008, Cohen’s *d* = 1.345) and BU-H (*p* = 0.037, Cohen’s *d* = 1.054) groups showed statistically significant attenuation of primary paw swelling by day 28 post-injection. Notably, the ET-H (*p* = 0.007, Cohen’s *d* = 1.393), EA-H (*p* = 0.004, Cohen’s *d* = 1.487), BU-H (*p* = 0.032, Cohen’s *d* = 1.095) and WA-H (*p* = 0.013, Cohen’s *d* = 1.279) groups achieved substantial inhibition of secondary paw swelling by day 28.

On the final day of treatment, the arthritis scores in the ET-H (*p* = 0.002, Cohen’s *d* = 1.580), PE-H (*p* = 0.005, Cohen’s *d* = 1.449), EA-H (*p* = 0.038, Cohen’s *d* = 1.054), BU-H (*p* = 0.005, Cohen’s *d* = 1.449), and WA-H (*p* = 0.002, Cohen’s *d* = 1.580) groups were significantly lower than those in the model group (Figure 3E). There were no significant differences in body weight among the different groups (Figure 3F). As shown in Figure 3G, the spleen index in the model group was significantly elevated compared to the control group (*p* = 0.018, Cohen’s *d* = 1.600). The WA-H group demonstrated a significantly lower spleen index relative to the model group (*p* = 0.047, Cohen’s *d* = 1.355).

Given that the effects of the ethanolic extract of *C. florida* on AA rats have been documented in previous studies, subsequent investigations focused on the EA, BU, and WA groups, which showed the most pronounced anti-IMPA effects.

#### 3.4.2 Histological analysis of AA rats

**Figure 4:**
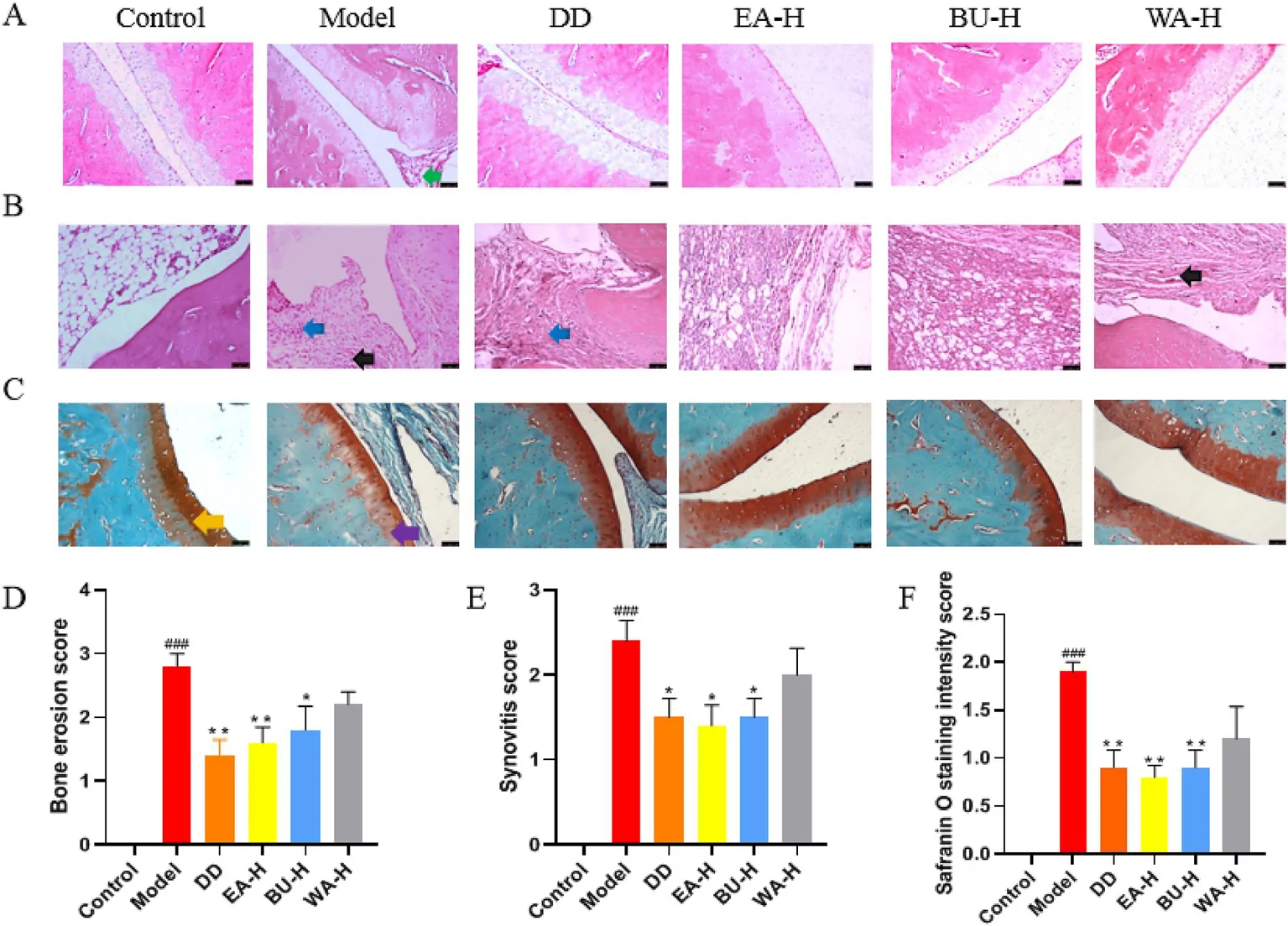
Histopathological images of the ankle joints in AA rats (*n* = 5 per group). **(A)** Osseous tissue section of AA rats. **(B)** Synovial tissue section of AA rats. **(C)** Histopathological Safranin O–fast green staining results of the ankle joints in AA rats. **(D)** Bone erosion score (0–3). **(E)** Synovitis score (0–3). **(F)** Safranin O staining intensity score (0–4). All histopathological figures were magnified by 200×. Scale bar: 50 μm. Vascular pannus formation (black arrow), synovial tissue hyperplasia (green arrow), inflammatory cell infiltration (blue arrow), chondrocytes (yellow arrow), and cartilage matrix (purple arrow). Data are presented as mean ± SEM. Statistical comparisons were performed using one-way ANOVA followed by Dunnett’s *post hoc* test. ^#^*p* < 0.05, ^##^*p* < 0.01, ^###^*p* < 0.001 vs. Control group; ^*^p < 0.05, ^**^p < 0.01, vs. model group.

As shown in Figures 4A–E, ankle joint tissues from rats in the control group exhibited no signs of inflammation or structural damage. The synovial lining was intact, with no evidence of inflammatory cell infiltration, consistent with normal joint architecture. In contrast, rats in the model group displayed severe pathological alterations: extensive inflammatory cell infiltration into the synovium and joint cavity, along with marked degradation of articular cartilage. Following treatment with *C. florida* fractions, these pathological features were ameliorated to varying degrees. Notably, the EA-H and BU-H groups demonstrated the most substantial improvements in joint morphology (all *p* < 0.05).

Safranin O–Fast Green staining (Figures 4C,F) further revealed that the cartilage in the control group maintained normal chondrocyte density and columnar organization, with smooth, continuous surfaces and uniform proteoglycan-rich matrix staining. The tidemark—the boundary between calcified and uncalcified cartilage—was clearly demarcated. In the model group, however, chondrocyte numbers were markedly reduced, cellular arrangement was disordered, and the cartilage surface appeared irregular and fibrillated. Proteoglycan content, as indicated by Safranin O staining, was significantly diminished, resulting in a pale, blurred matrix appearance and loss of a distinct tidemark.

In comparison, the EA-H (*p* = 0.002, Cohen’s *d* = 2.630) group showed partial restoration of chondrocyte density, smoother cartilage surfaces, a more discernible tidemark, and near-normal Safranin O staining intensity—suggesting preserved proteoglycan content. Similarly, the BU-H (*p* = 0.004, Cohen’s *d* = 2.391) group exhibited increased chondrocyte numbers, improved cellular alignment, reduced surface irregularities, and enhanced matrix staining relative to the model group. Collectively, these findings indicate that *C. florida*-derived fractions, particularly EA-H and BU-H, effectively attenuated cartilage destruction and synovial inflammation in adjuvant-induced arthritic rats, thereby preserving joint integrity.

#### 3.4.3 Effects of *C. florida* fractions on the expression levels of RF, IL-6, TNF-α, and COX-2 in the serum of AA rats

**Figure 5:**
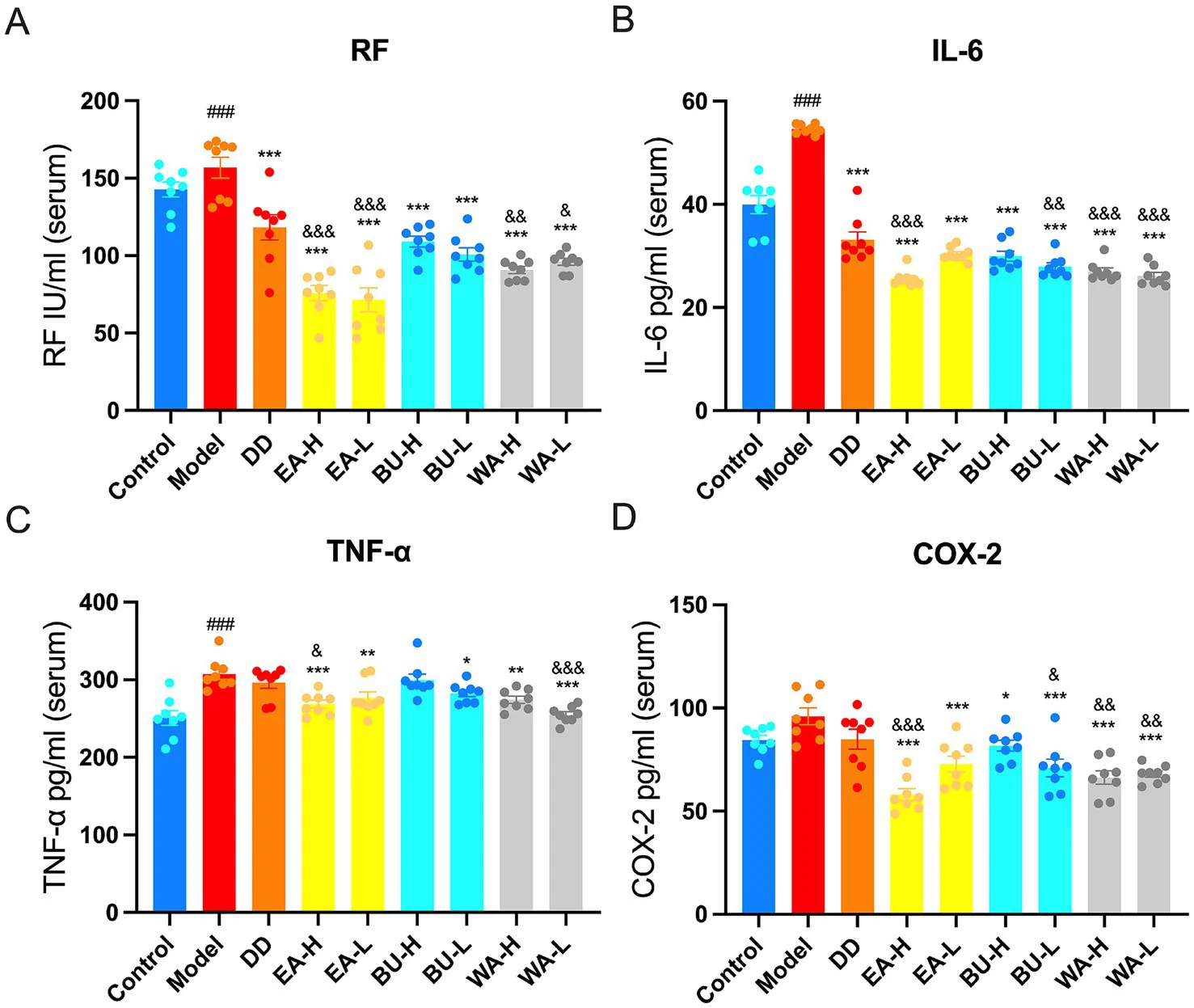
Effects of *C. florida* fractions on serum inflammation markers in AA rats (*n* = 8 per group). **(A)** Serum RF level. **(B)** Serum IL-6 level. **(C)** Serum TNF-α level. **(D)** Serum COX-2 level. Data are presented as mean ± SEM. Statistical comparisons were performed using one-way ANOVA followed by Dunnett’s *post hoc* test. ^###^*p* < 0.001 vs. control group; ^*^p < 0.05, ^**^p < 0.01, ^***^p < 0.001 vs. model group; ^&^*p* < 0.05, ^&&^*p* < 0.01, ^&&&^*p* < 0.001 vs. DD group.

Compared with the control group, serum levels of IL-6 and TNF-α were significantly elevated in the model group (all *p* < 0.001; see Supplementary Table S4–S7 for exact *p* values and effect sizes), confirming systemic inflammation in CFA-induced arthritis (Figure 5). Treatment with *C. florida* fractions markedly attenuated this pro-inflammatory response. Specifically, both the EA-H and WA-H groups exhibited significantly lower serum concentrations of TNF-α, COX-2, IL-6, and RF compared to the model group (all *p* < 0.001).

Notably, the EA-H and WA-L groups exhibited significantly greater efficacy than the positive control group, as evidenced by markedly lower levels of TNF-α, COX-2, IL-6, and RF (all *p* < 0.05). These findings suggest that *C. florida* fraction—particularly WA fractions—not only effectively suppress key inflammatory mediators but may also exhibit superior efficacy to the positive control (diclofenac) in modulating systemic immune markers. Collectively, the data indicate that topical application of *C. florida* fractions potently attenuates the inflammatory cascade associated with CFA-induced arthritis.

#### 3.4.4 The ERK/MAPK and NF-κB signaling pathways may be involved in the anti-IMPA mechanism of *C. florida* fractions

**Figure 6:**
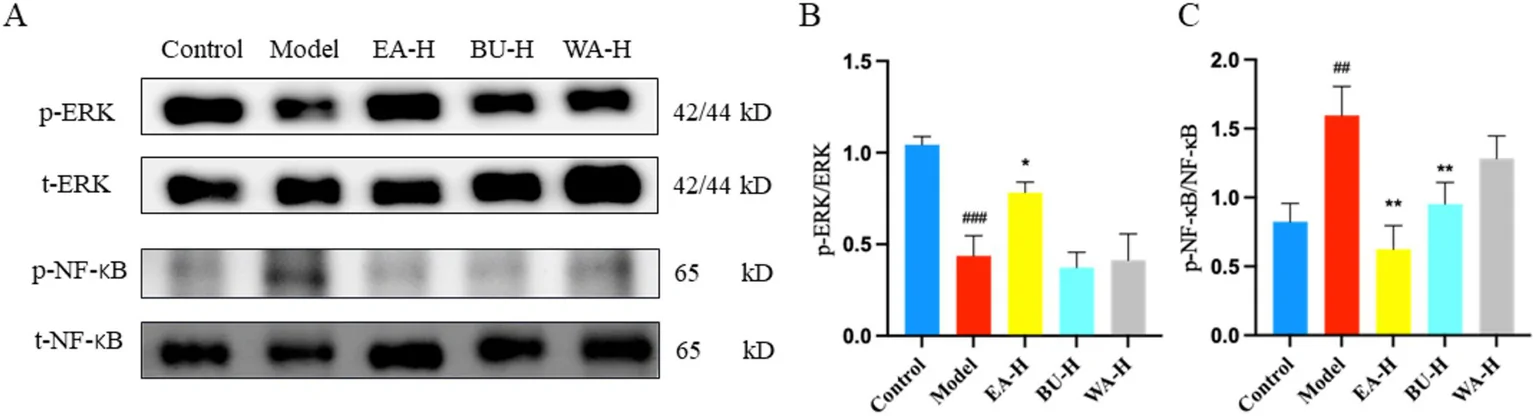
Effect of *C. florida* fractions on the phosphorylation of ERK1/2 and NF-κB p65 in AA rats (*n* = 3 per group). **(A)** Representative Western blot images showing phospho-ERK1/2, total ERK1/2, phospho-NF-κB p65, and total NF-κB p65 in synovial tissues from different experimental groups. **(B)** Quantification of the phospho-ERK1/2 to total ERK1/2 ratio. **(C)** Quantification of the phospho-NF-κB p65 to total NF-κB p65 ratio. Data are presented as mean ± SEM. Statistical significance was determined by one-way ANOVA followed by Dunnett’s *post hoc* test. ^##^*p* < 0.01, ^###^*p* < 0.001 vs. control group; *p < 0.05, ^**^p < 0.01 vs. model group.

To determine whether the anti-arthritic effects of *C. florida* fractions in AA rats are mediated through the ERK/MAPK and NF-κB signaling pathways, we assessed the phosphorylation status of ERK1/2 and NF-κB p65 in knee synovial tissue via Western blot analysis (Figure 6). Compared to the control group, the model group showed a significant decrease in p-ERK1/2 levels (*p* = 0.001, Cohen’s *d* = 3.698), which was effectively restored to near-basal levels following treatment with the EA fraction of *C. florida* (*p* = 0.028, Cohen’s *d* = 2.095). Conversely, phosphorylation of NF-κB p65 was markedly upregulated in the model group (*p* = 0.002, Cohen’s *d* = 2.635) and was significantly attenuated by both the EA (*p* = 0.002, Cohen’s *d* = 3.317) and BU (*p* = 0.023, Cohen’s *d* = 2.197) fraction of *C. florida*.

**Figure 7:**
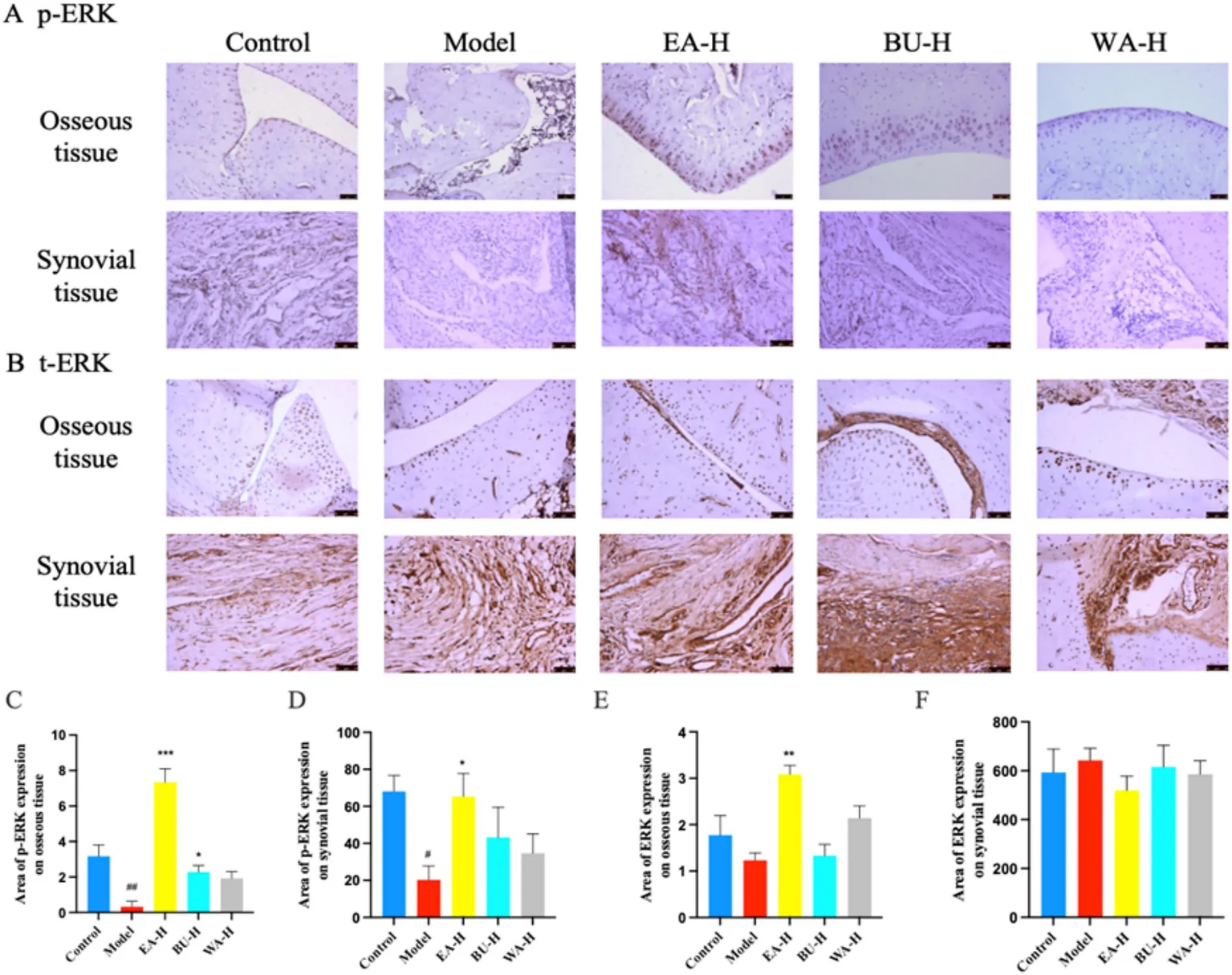
Immunohistochemical staining of ERK1/2 proteins in AA rats. **(A)** Immunohistochemical staining of p-ERK1/2 expression. **(B)** Immunohistochemical staining of total ERK1/2 expression. **(C)** Area of p-ERK1/2 expression on osseous tissue. **(D)** Area of p-ERK1/2 expression on synovial tissue. **(E)** Area of total ERK1/2 expression on osseous tissue. **(F)** Area of total ERK1/2 expression on synovial tissue (*n* = 5). All figures were magnified by 200×. Scale: 50 μm. Statistical comparisons were performed using one-way ANOVA followed by Dunnett’s *post hoc* test. ^#^*p* < 0.05, ^##^*p* < 0.01 vs. control group ^*^p < 0.05, ^**^p < 0.01, ^***^p < 0.001 vs. model group.

**Figure 8:**
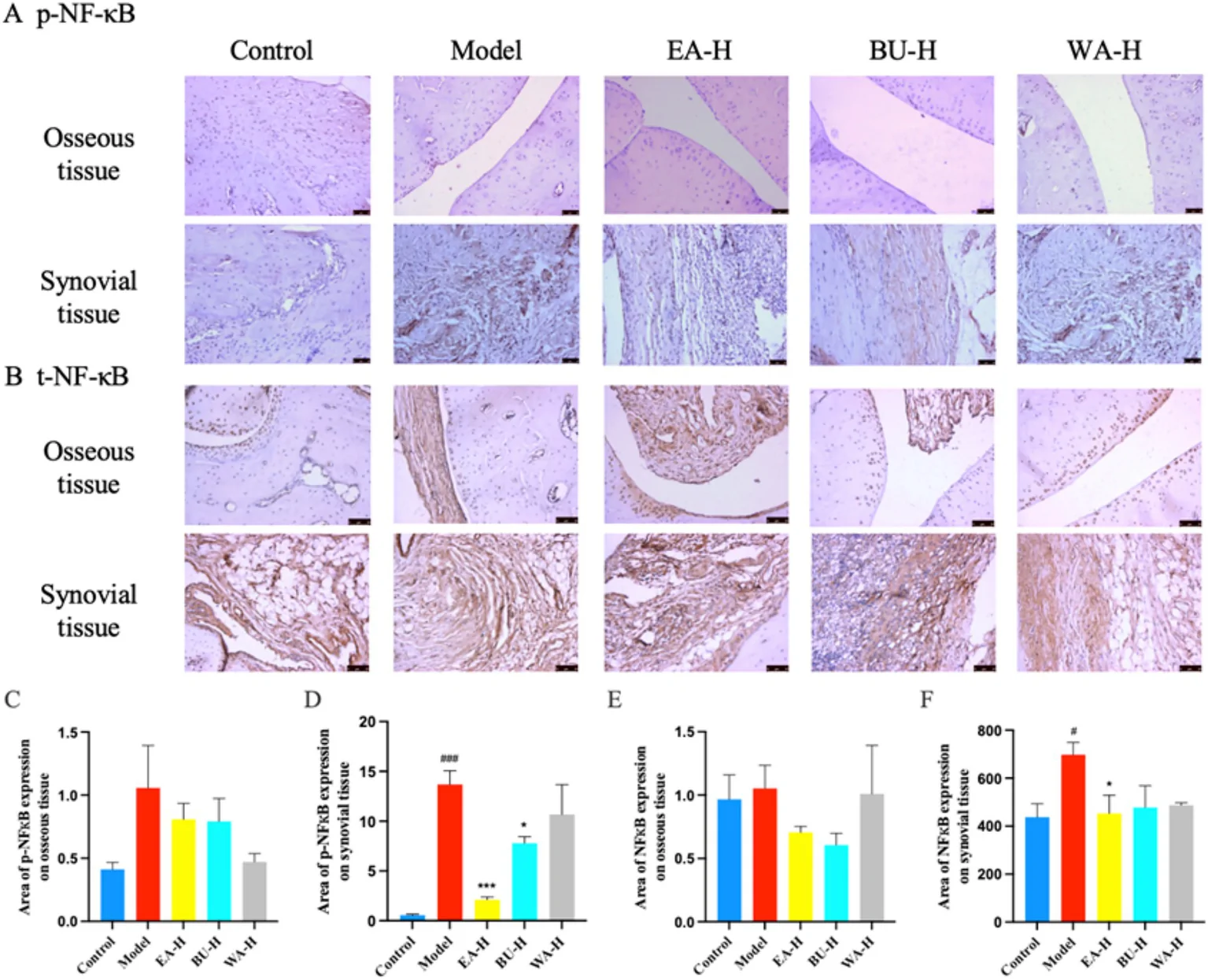
Immunohistochemical staining of NF-κB p65 proteins in AA rats. **(A)** Immunohistochemical staining of p-NF-κB p65 expression. **(B)** Immunohistochemical staining of total NF-κB p65 expression. **(C)** Area of p-NF-κB p65 expression on osseous tissue. **(D)** Area of p-NF-κB p65 expression on synovial tissue. **(E)** Area of total NF-κB p65 expression on osseous tissue. **(F)** Area of total NF-κB p65 expression on synovial tissue (*n* = 5). All figures were magnified by 200×. Scale bar: 50 μm. Statistical comparisons were performed using one-way ANOVA followed by Dunnett’s *post hoc* test. ^#^*p* < 0.05, ^###^*p* < 0.001 vs. Mcontrol group; ^*^p < 0.05, ^**^*p < 0.001 vs. model group.

To further localize these molecular alterations, immunohistochemical analysis was performed on ankle joint sections (Figures 7, 8). As illustrated in Figure 7, p-ERK1/2 immunoreactivity was predominantly observed in articular chondrocytes. Consistent with the Western blot results, p-ERK1/2 expression was markedly reduced in the model group compared to controls but was significantly restored in the EA-treated group. In contrast, p-NF-κB p65 staining was barely detectable in control tissues yet strongly upregulated in both synovial and cartilage compartments of model rats (Figure 8). This pathological hyperactivation was effectively suppressed following treatment with *C. florida* fractions, most notably in the EA and BU groups.

Collectively, after 28 days of treatment, *C. florida* fractions—especially the ethyl acetate fraction—significantly enhanced ERK1/2 phosphorylation while inhibiting NF-κB p65 activation in joint tissues relative to the model group. These findings suggest that the anti-arthritic effects of *C. florida* may be associated with the modulation of the ERK/MAPK and NF-κB signaling pathways, specifically by promoting protective ERK signaling and suppressing pro-inflammatory NF-κB activation.

## 4 Discussion

*C. florida*, a medicinal plant traditionally used in East Asia, has been reported to possess anti-inflammatory properties; however, its pharmacological effects following topical administration have not been systematically investigated (14). Despite its long-standing use, research on *C. florida* remains limited, and the mechanisms underlying the topical anti-inflammatory, analgesic, and anti-edema effects of its polarity-based fractions have not been elucidated. To the best of our knowledge, this study provides the first systematic *in vivo* evaluation of the analgesic, anti-inflammatory, and anti-edema activities of topically applied *C. florida* fractions—specifically, the PE, EA, BU, and WA fractions—alongside a mechanistic analysis of their therapeutic actions.

The xylene-induced ear edema model was employed to assess the anti-inflammatory potential of *C. florida* fractions. This model triggers the release of pro-inflammatory mediators and is a well-established method for preliminary screening of topical anti-inflammatory agents. It provides a rapid and reproducible assessment of acute inflammatory responses, allowing quantitative evaluation of topical efficacy (30). The model is particularly relevant as the inflammation is confined to the application site. DD, a clinically used topical nonsteroidal anti-inflammatory drug (NSAID), was included as a positive control because it is standard for treating local inflammatory symptoms such as swelling and pain. Topical corticosteroids were excluded due to their limited skin penetration and risk of local side effects in peripheral joint conditions (31). Topical application of all *C. florida* fractions significantly attenuated xylene-induced ear swelling in a dose-dependent manner. Notably, the medium-dose BU fraction and low-dose WA fraction exhibited the strongest inhibitory effects, suggesting that these fractions may exert anti-inflammatory activity by suppressing the release or action of inflammatory mediators *in vivo*.

The acetic acid–induced abdominal writhing test in mice—a well-established model of chemical nociception—was used to evaluate peripheral analgesic effects. Intraperitoneal acetic acid induces peritoneal irritation and tissue injury, triggering the release of inflammatory and algesic mediators that activate peripheral nociceptors (32, 33). Pain intensity is quantified by counting writhing episodes. In this study, topical treatment with *C. florida* fractions significantly reduced writhing responses compared to controls. The medium-dose EA fraction, high-dose BU fraction, and high-dose aqueous fraction showed the most potent analgesic effects, indicating a peripheral mechanism likely associated with suppression of inflammation-related nociceptive mediators.

The CFA-induced arthritis model in rats is a widely accepted experimental paradigm for RA, characterized by an immune complex–mediated inflammatory response (34). Intradermal injection of CFA delivers a sustained immunogenic stimulus, eliciting a robust secondary autoimmune reaction that mirrors key features of IMPA. Given that the core pathological feature of RA is chronic synovitis driven by aberrant immune activation, this study employed the AA rat model to evaluate the therapeutic efficacy of polar fractions of *C. florida* against IMPA. A single intradermal injection of CFA was administered to induce arthritis, resulting in pronounced swelling in both primary (injected) and secondary (contralateral and distal) joints in the model group—consistent with the systemic polyarticular pattern typical of IMPA. Although repeated CFA injections can produce a more severe and chronic phenotype, a single-dose protocol was selected herein to align with the topical route of drug administration and to maintain consistency with established methodological precedents in the literature (35).

Our experimental findings demonstrated that *C. florida* fractions dose-dependently attenuated paw swelling and reduced clinical arthritis scores in AA rats. Notably, both the EA and WA fractions significantly decreased the spleen index in AA rats, suggesting that these effects may reflect a secondary attenuation of systemic inflammatory burden resulting from effective local control of joint inflammation. Histopathological analysis of ankle joints further confirmed the protective actions of *C. florida* fractions: treated animals exhibited markedly reduced synovial hyperplasia and diminished inflammatory cell infiltration compared to the model control group. Moreover, *C. florida* treatment preserved articular cartilage integrity, as evidenced by higher chondrocyte density and well-maintained tidemarks in joint sections. Importantly, no significant body weight loss was observed in AA rats throughout the study, likely attributable to the low-dose CFA protocol and the non-invasive nature of topical drug administration, which avoided gastrointestinal irritation and supported overall animal well being.

Our research group previously reported an oral LD_50_ of 60.08 g/kg for the aqueous extract of *C. florida* in mice, indicating a relatively high acute toxicity threshold via the oral route. However, emerging evidence suggests that certain *C. florida* extracts may exert developmental toxicity in zebrafish embryos, underscoring the need for careful dose optimization and patient selection when considering oral administration (36). In contrast, our prior studies have demonstrated that topical application of the *C. florida* ethanol extract is well tolerated, with no observable local or systemic toxicity or adverse reactions, supporting its safety as a dermal therapeutic agent (37). Topical administration may offer a favorable safety profile compared with oral delivery, particularly for chronic inflammatory conditions such as IMPA. In this study, the topical formulation was prepared as an aqueous suspension. We hypothesize that dermal absorption of the active constituents is primarily facilitated by the hydration effect of the vehicle in conjunction with the polarity-based fractionation of the *C. florida* extracts. Furthermore, we propose that the undissolved solid particles in the formulation create a reservoir effect at the application site, maintaining a localized concentration gradient to sustain anti-inflammatory activity against xylene-induced acute edema.

The phytochemical analysis revealed that flavonoids and saponins—both known for their anti-inflammatory and immunomodulatory properties—were most abundant in the WA fraction, followed by the BU and EA fractions, while the PE fraction containing the lowest levels. This distribution pattern closely aligns with the observed pharmacological activities: the WA, BU, and EA fractions demonstrated significantly stronger anti-inflammatory, analgesic, and therapeutic effects in the adjuvant-induced arthritis model compared to the PE fraction. Given that saponins are highly polar glycosides and many flavonoids also occur in glycosidic forms, their preferential solubility in aqueous and moderately polar solvents explains their enrichment in these active fractions. These findings strongly indicate that the efficacy of *C. florida* is largely attributable to its polar constituents.

In a previous study, our research group conducted a network pharmacology analysis to explore the potential molecular targets and signaling pathways associated with *C. florida*. This analysis identified ERK and NF-κB as key signaling pathways potentially involved in its pharmacological actions of *C. florida*. Subsequent experimental validation using the total ethanol extract supported the involvement of these pathways in mediating anti-inflammatory effects (16). Based on these prior findings, the present study was designed to further evaluate whether polarity-based fractions of *C. florida*, administered topically, exert anti-arthritic effects through modulation of ERK- and NF-κB–related signaling *in vivo*.

To further explore the molecular mechanisms underlying the protective effects of *C. florida* fractions in adjuvant-induced arthritis, we examined the activation status of ERK in joint tissues. ERK signaling is widely recognized as an important regulator of cellular responses involved in inflammatory joint diseases, including synovial proliferation, chondrocyte survival, and cartilage homeostasis. ERK activation has been reported to participate in tissue repair processes and may contribute to the maintenance of articular cartilage integrity under inflammatory conditions (38, 39). In the present study, treatment with the EA fraction of *C. florida* significantly increased the p-ERK/total ERK ratio in ankle joint tissues. Immunohistochemical analysis further demonstrated that phosphorylated ERK was predominantly localized in chondrocytes within the articular cartilage. Although ERK signaling in arthritis is complex and context-dependent, transient or modulated ERK activation has been previously associated with tissue repair and chondroprotection (40). Therefore, we propose that the extract may modulate this pathway, contributing to the observed therapeutic effects, though further studies are needed to fully elucidate the precise mechanisms.

As predicted by network pharmacology and validated in prior studies, NF-κB signaling was examined as a key inflammatory pathway in this study. It serves as a central regulator of inflammatory responses and plays a critical role in the initiation and progression of IMPA by promoting the transcription of pro-inflammatory mediators, including TNF-α, COX-2, and IL-6 (41). Persistent activation of NF-κB contributes to synovial inflammation, pannus formation, and progressive joint destruction, making this pathway a key pharmacological target in IMPA management (42). In the present study, serum levels of RF, TNF-α, and IL-6, as well as COX-2 expression, were markedly elevated in adjuvant-induced arthritis rats, consistent with enhanced inflammatory activity. This suggests that effective mitigation of local joint inflammation subsequently reduces the systemic inflammatory burden. Topical treatment with polarity-based fractions of *C. florida*, particularly the EA and BU fractions, significantly attenuated these inflammatory markers. Furthermore, both Western blot and immunohistochemical analyses demonstrated that these fractions effectively suppressed NF-κB activation, as evidenced by reduced levels of phosphorylated NF-κB in synovial and ankle joint tissues. Collectively, these findings indicate that inhibition of NF-κB–related signaling is closely associated with the anti-inflammatory and anti-arthritic effects of *C. florida* fractions following topical administration. By downregulating NF-κB activation and its downstream pro-inflammatory mediators, *C. florida* fractions may alleviate synovial inflammation and joint pathology in experimental arthritis. Nevertheless, the precise molecular interactions between *C. florida*-derived constituents and components of the NF-κB pathway remain to be elucidated and warrant further investigation.

Our findings demonstrate that the topical application of *C. florida* exerts significant therapeutic effects in the CFA-induced arthritis model through a dual modulatory mechanism: inhibition of the pro-inflammatory NF-κB pathway and activation of the protective ERK signaling pathway. This multi-target action contrasts with conventional topical NSAIDs, such as DD gel, which primarily act by inhibiting cyclooxygenase (COX) enzymes and downstream prostaglandin synthesis (43). By simultaneously dampening destructive inflammation (via NF-κB suppression) and promoting tissue resilience (via ERK-mediated chondroprotection), *C. florida* fractions may offer a more holistic approach to managing immune-mediated polyarthritis. In the context of veterinary medicine, this topical approach represents a potential strategy that warrants further investigation, but also faces unique challenges. While systemic NSAIDs are effective, their use in dogs carries well-documented risks of gastrointestinal and renal toxicity. Topical formulations are designed to minimize systemic exposure and thus mitigate these risks; however, their practical application in dogs is complicated by behaviors such as self-trauma from licking the application site and uncertainties regarding joint bioavailability (44, 45). Furthermore, species within the genus *Clematis*, including *C. florida*, have a long history of traditional use in Chinese medicine for treating joint disorders, with phytochemical studies suggesting the presence of flavonoids and saponins possessing general anti-inflammatory properties (14, 46).

Critically, our preliminary safety assessment confirmed that the *C. florida* formulation is non-irritating to the skin, indicating excellent local tolerability (37). This favorable profile not only addresses a key concern for topical therapies but also suggests it may circumvent the local adverse effects associated with other agents (e.g., the burning sensation from capsaicin) and the systemic toxicities of oral NSAIDs. Whether *C. florida* ultimately offers a superior safety and efficacy profile compared to existing options remains to be determined. Therefore, future studies should include direct head-to-head comparisons with standard topical NSAIDs, detailed toxicological evaluations (including assessments of potential ingestion due to licking), and further mechanistic investigations to fully clarify its therapeutic potential and risk–benefit ratio for companion animals.

While this study provides valuable insights into the therapeutic efficacy of the topical application of *C. florida*, several limitations should be acknowledged. First, given that topical administration is central to this research, the absence of direct pharmacokinetic (PK) or skin permeation data is noted. While the significant improvement in local symptoms suggests effective local delivery, we did not quantify local tissue exposure via *in vivo* dermal penetration assays. Future studies will incorporate advanced non-invasive imaging or local PK evaluations to further elucidate the transdermal behavior of specific compounds. Second, it is important to note that all experiments were conducted using murine models. While widely used for screening anti-arthritic agents, the pathophysiology of induced arthritis in rodents differs from naturally occurring immune-mediated polyarthritis in companion animals. Therefore, the potential veterinary applications discussed herein should be interpreted with caution, and future studies in target species are needed to validate these translational findings. Third, we acknowledge that the Western blot analyses were conducted with a limited number of biological replicates due to practical constraints such as gel capacity and tissue availability. Although rigorous normalization and consistency with *in vivo* phenotypic data support the reliability of our results, the limited sample size may impact the statistical power of the mechanistic conclusions. Finally, regarding the mechanistic conclusions, our current findings demonstrate an association between the therapeutic effects and the modulation of ERK and NF-κB signaling, rather than definitive causality. The absence of specific pathway inhibitors or genetic knockouts precludes definitive causal inference. Therefore, these signaling pathways should be interpreted as key correlates of the observed efficacy, and future studies employing targeted molecular interventions are warranted to establish a definitive causal link.

## 5 Conclusion

In summary, the present study demonstrates that polarity-based fractions of *C. florida* exert significant anti-inflammatory, analgesic, and anti-arthritic effects following topical administration in established animal models. Among the tested fractions, the ethyl acetate, *n*-butanol, and aqueous fractions exhibited particularly pronounced pharmacological efficacy, as evidenced by the attenuation of joint swelling, improvement of histopathological alterations, and suppression of pro-inflammatory mediators. Mechanistic investigations further indicated that these protective effects are closely associated with modulation of ERK- and NF-κB–related signaling pathways in joint tissues, suggesting a potential role of these pathways in mediating the local therapeutic actions of *C. florida* fractions.

From a pharmacological perspective, topical delivery represents a rational and clinically relevant approach for the management of IMPA, as it allows direct targeting of inflamed joints while potentially minimizing systemic exposure and adverse effects commonly associated with long-term oral anti-arthritic therapies. The favorable efficacy observed in this study, together with the absence of apparent treatment-related toxicity, supports the feasibility of developing *C. florida*–derived topical preparations as complementary agents for inflammatory joint diseases.

Collectively, these findings provide pharmacological evidence supporting the topical anti-arthritic potential of *C. florida* fractions and offer a scientific basis for further development of plant-derived topical therapeutics for the management of IMPA.

## Data Availability

The original contributions presented in the study are included in the article/Supplementary material, further inquiries can be directed to the corresponding author/s.
