# Supplementary material for: Anti-inflammatory, analgesic, and anti-immune-mediated polyarthritis activities of topically applied polarity fractions of Clematis florida var. Plena and their underlying mechanisms
Source: Front Vet Sci. 2026 Aug 26;13:1846520. doi: 10.3389/fvets.2026.1846520 (PMC13553389; doi:10.3389/fvets.2026.1846520)
Supplement: Supplementary file 1 [file Data_Sheet_1.DOCX]

Figure S1. Total ion chromatograms (TICs) of C. florida extracts by mass spectrometry. (A) PE; (B) EA; (C) BU; (D) WA. The left-hand panels display the positive ion mode, while the right-hand panels show the negative ion mode.

Table S1. Identification of chemical constituents in the *C. florida* extracts by UPLC-MS/HRMS

| No | m/z | RT (min) | Adduct | Compound name | Formula | Peak area | | | |
| --- | --- | --- | --- | --- | --- | --- | --- | --- | --- |
|  |  |  |  |  |  | WA | BU | EA | PE |
| 1 | 182.0812 | 0.82 | [M+H]^+^ | L-(-)-Tyrosine | C_9_H_11_NO_3_ | / | / | / | 2.73×10^8^ |
| 2 | 169.013 | 1.06 | [M-H]^-^ | Gallic acid | C_7_H_6_O_5_ | / | / | 1.10×10^7^ | / |
| 3 | 166.0862 | 1.07 | [M+H]^+^ | L-(-)-Phenylalanine | C_9_H_11_NO_2_ | / | / | / | 2.90×10^9^ |
| 4 | 153.0182 | 1.8 | [M-H]^-^ | Protocatechuic acid | C_7_H_6_O_4_ | / | / | 5.15×10^8^ | / |
| 5 | 205.0971 | 2.07 | [M+H]^+^ | L-(-)-Tryptophan | C_11_H_12_N_2_O_2_ | / | / | / | 1.23×10^9^ |
| 6 | 137.0232 | 2.55 | [M-H]^-^ | 4-Hydroxybenzoic acid | C_7_H_6_O_3_ | 5.64×10^5^ | 5.66×10^6^ | 2.42×10^9^ | 1.92×10^7^ |
| 7 | 192.0768 | 2.83 | [M+H]^+^ | Carbendazim | C_9_H_9_N_3_O_2_ | / | 1.94×10^6^ | 1.26×10^8^ | / |
| 8 | 197.1284 | 3.2 | [M+H]^+^ | Cyclo(proline-valine) | C_10_H_16_N_2_O_2_ | / | 1.15×10^8^ | 1.58×10^8^ | / |
| 9 | 169.0495 | 3.82 | [M+H]^+^ | Vanillic acid | C_8_H_8_O_4_ | / | / | 2.76×10^8^ | / |
| 10 | 325.0555 | 4.1 | [M-H]^-^ | Fertaric acid | C_14_H_14_O_9_ | / | 5.82×10^4^ | 6.13×10^8^ | 1.28×10^8^ |
| 11 | 179.0339 | 4.16 | [M-H]^-^ | Caffeic acid | C_9_H_8_O_4_ | / | / | 3.29×10^9^ | 2.55×10^7^ |
| 12 | 371.1335 | 4.35 | [M-H]^-^ | Eleutheroside B | C_17_H_24_O_9_ | / | / | 7.20×10^6^ | 2.31×10^7^ |
| 13 | 199.06 | 4.47 | [M+H]^+^ | Syringic acid | C_9_H_10_O_5_ | / | / | 3.21×10^8^ | / |
| 14 | 342.1702 | 4.47 | [M+H]^+^ | Fuzitine | C_20_H_24_NO_4_ | / | / | / | 2.25×10^7^ |
| 15 | 165.0911 | 4.84 | [M+H]^+^ | Isoeugenol | C_10_H_12_O_2_ | 9.03×10^7^ | 5.07×10^8^ | 1.25×10^9^ | 1.73×10^8^ |
| 16 | 257.1381 | 4.87 | [M+H]^+^ | Lawsorosemarinol | C_13_H_20_O_5_ | 2.07×10^8^ | 1.05×10^9^ | 2.80×10^9^ | 1.53×10^8^ |
| 17 | 256.1653 | 5.59 | [M+H]^+^ | L-Pyrrolsine | C_12_H_21_N_3_O_3_ | / | / | / | 1.14×10^9^ |
| 18 | 299.2075 | 5.62 | [M+H]^+^ | Pareptide | C_14_H_26_N_4_O_3_ | / | / | / | 4.90×10^8^ |
| 19 | 521.2009 | 5.73 | [M-H]^-^ | Lariciresinol 9-O-β -D-glucopyranoside | C_26_H_34_O_11_ | / | 4.75×10^5^ | / | 9.99×10^6^ |
| 20 | 163.0389 | 5.83 | [M-H]^-^ | p-Coumaric acid | C_9_H_8_O_3_ | / | / | 2.71×10^8^ | / |
| 21 | 525.1956 | 5.9 | [M-H]^-^ | Threo-4,7,9,9′-tetrahydroxy-3-methoxy-8-O-4′-neolignan 3′-O-β-D-glucopyranoside | C_25_H_34_O_12_ | / | / | / | 3.54×10^6^ |
| 22 | 539.2117 | 6.7 | [M-H]^-^ | Threo-7,9,9′-trihydroxy-3,3′-dimethoxy-8-O-4′-neolignan 4-O-β-D-glucopyranoside | C_26_H_36_O_12_ | / | 1.10×10^6^ | 3.61×10^6^ | 1.42×10^7^ |
| 23 | 245.1283 | 6.99 | [M+H]^+^ | Cyclo(phenylalanine-proline) | C_14_H_16_N_2_O_2_ | / | 1.03×10^8^ | 9.76×10^7^ | / |
| 24 | 331.1025 | 7.07 | [M-H]^-^ | Leonuriside A | C_14_H20O9 | / | / | 3.51×10^7^ | 2.59×10^5^ |
| 25 | 539.2117 | 7.27 | [M-H]^-^ | Threo-7,9,9′-trihydroxy-3,3′-dimethoxy-8-O-4′-neolignan 4-O-β-D-glucopyranoside | C_26_H_36_O_12_ | / | 6.37×10^5^ | 1.99×10^6^ | 9.11×10^6^ |
| 26 | 521.2009 | 7.99 | [M-H]^-^ | Lariciresinol 4-O-β -D-glucopyranoside/Lariciresinol 4'-O-β-D-glucopyranoside | C_26H34_O_11_ | / | 2.92×10^6^ | 9.08×10^6^ | 1.77×10^7^ |
| 27 | 551.2113 | 8.16 | [M-H]^-^ | Alangilignoside D/Forsythianoside B/5'-Methoxyisolariciresinol 9'-O-β-D-glucopyranoside/ | C_27_H_36_O_12_ | / | 1.77×10^5^ | / | 2.48×10^6^ |
| 28 | 367.1387 | 8.68 | [M-H]^-^ | Hexapetoside C | C_18_H_24_O_8_ | 1.72×10^5^ | 3.42×10^7^ | 3.76×10^7^ | 9.91×10^6^ |
| 29 | 377.1592 | 8.68 | [M-H]^-^ | Threo-4,7,9,9'-tetrahydroxy-3,3'-dimethoxy-8-O-4'-neolignan | C20H26O7 | 1.85×10^7^ | 1.86×10^8^ | 6.54×10^7^ | 7.04×10^6^ |
| 30 | 193.0496 | 8.77 | [M-H]^-^ | Ferulic acid/Isoferulic acid | C_10_H_10_O_4_ | / | / | 1.40×10^9^ | 2.60×10^8^ |
| 31 | 521.2009 | 10.31 | [M-H]^-^ | Lariciresinol 9-O-β -D-glucopyranoside | C_26_H_34_O_11_ | 6.60×10^4^ | 2.09×10^6^ | 1.62×10^7^ | 5.97×10^6^ |
| 32 | 519.1852 | 10.54 | [M-H]^-^ | Pinoresinol 4-O-β -D-glucopyranoside/Pinoresinol 4'-O-β-D-glucopyranoside | C_26_H_32_O_11_ | 6.89×10^5^ | 5.08×10^6^ | 1.41×10^7^ | 1.32×10^7^ |
| 33 | 521.2009 | 11.01 | [M-H]^-^ | Lariciresinol 9-O-β -D-glucopyranoside/Isolariciresinol 9′-O-β -D-glucopyranosidee | C_26_H_34_O_11_ | 1.75×10^5^ | 8.02×10^6^ | 2.24×10^7^ | 2.05×10^7^ |
| 34 | 193.0859 | 11.33 | [M+H]^+^ | 4-Methoxy-6-(prop-2-en-1-yl)-2H-1,3-benzodioxole | C_11_H_12_O_3_ | / | 3.89×10^7^ | 1.01×10^8^ | / |
| 35 | 551.2113 | 11.53 | [M-H]^-^ | Alangilignoside D/Forsythianoside | C_27_H_36_O_12_ | / | 2.04×10^5^ | / | 9.85×10^5^ |
| 36 | 521.2009 | 11.59 | [M-H]^-^ | Lariciresinol 9-O-β -D-glucopyranoside/Isolariciresinol 9′-O-β -D-glucopyranoside | C_26_H_34_O_11_ | / | 7.74×10^5^ | / | 2.62×10^6^ |
| 37 | 355.1385 | 12.44 | [M-H]^-^ | 4-Allyl-2,6-dimethoxyphenylglucoside | C_17_H_24_O_8_ | / | / | 2.96×10^7^ | / |
| 38 | 249.1482 | 13 | [M+H]^+^ | 11β,13-Dihydrozaluzanin C | C_15_H_20_O_3_ | / | 1.50×10^7^ | 2.08×10^7^ | / |
| 39 | 537.1952 | 15.1 | [M-H]^-^ | Lanicepside A/Lanicepside B | C_26_H_34_O_12_ | / | / | 8.10×10^6^ | / |
| 40 | 531.1484 | 17.27 | [M-H]^-^ | Hexapetoside H | C_26_H_28_O_12_ | / | / | 1.20×10^7^ | / |
| 41 | 327.2166 | 20.74 | [M-H]^-^ | Corchorifatty acid F | C_18_H_32_O_5_ | 1.20×10^7^ | 5.12×10^6^ | 2.15×10^9^ | 4.91×10^7^ |
| 42 | 495.149 | 20.93 | [M-H]^-^ | 4'-O-[6"-O-(4"-Hydroxy-3",5"-dimethoxybenzoyl)-β-D-glucopyranosyl]-3'-hydroxyphenethyl alcohol | C_23_H_28_O_12_ | / | / | 2.60×10^7^ | 1.15×10^6^ |
| 43 | 327.2166 | 21.05 | [M-H]^-^ | Corechorifatty acid F | C_18_H_32_O_5_ | 1.64×10^7^ | 3.90×10^6^ | 2.11×10^9^ | 5.09×10^7^ |
| 44 | 407.1728 | 21.55 | [M-H]^-^ | Glucozaluzanin C | C_21_H_28_O_8_ | / | / | 1.71×10^8^ | 2.91×10^7^ |
| 45 | 375.1279 | 22.36 | [M+H]^+^ | Methyl syringate 4-O-β -D-glucopyranoside | C_16_H_22_O_10_ | / | / | 2.04×10^6^ | 1.20×10^7^ |
| 46 | 389.1437 | 22.44 | [M+H]^+^ | Hexapetoside I | C_17_H_24_O_10_ | / | / | 5.60×10^6^ | 2.20×10^7^ |
| 47 | 285.0754 | 23.82 | [M+H]^+^ | Wogonin | C_16_H_12_O_5_ | / | 1.61×10^7^ | / | / |
| 48 | 403.1383 | 24.21 | [M+H]^+^ | Nobiletin | C_21_H_22_O_8_ | / | 1.43×10^6^ | / | 3.25×10^6^ |

Table S2. Pairwise comparisons of ear edema between drug-treated groups and the control group in the xylene-induced ear edema: *p* values, and Cohen’s *d* effect sizes

| Dunnett's multiple comparisons test | Adjusted *P* value | Cohen’s *d* |
| --- | --- | --- |
| Control vs. DD | <0.001 | 3.364 |
| Control vs. ET-H | <0.001 | 2.935 |
| Control vs. ET-L | <0.001 | 2.124 |
| Control vs. PE-H | <0.001 | 2.227 |
| Control vs. PE-M | <0.001 | 2.161 |
| Control vs. PE-L | 0.002 | 1.938 |
| Control vs. EA-H | <0.001 | 2.581 |
| Control vs. EA-M | <0.001 | 2.078 |
| Control vs. EA-L | <0.001 | 2.637 |
| Control vs. BU-H | <0.001 | 2.917 |
| Control vs. BU-M | <0.001 | 3.410 |
| Control vs. BU-L | <0.000 | 2.153 |
| Control vs. WA-H | 0.036 | 1.500 |
| Control vs. WA-M | <0.001 | 2.217 |
| Control vs. WA-L | <0.001 | 3.801 |

Table S3. Pairwise comparisons of writhing responses between drug-treated groups and the control group in the acetic acid-induced writhing test: *p* values, and Cohen’s *d* effect sizes

| Dunnett's multiple comparisons test | Adjusted *P* value | Cohen’s *d* |
| --- | --- | --- |
| Control vs. DD | <0.001 | 2.680 |
| Control vs. ET-H | <0.001 | 3.058 |
| Control vs. ET-L | 0.976 | 0.489 |
| Control vs. PE-H | 0.006 | 1.790 |
| Control vs. PE-M | 0.372 | 0.978 |
| Control vs. PE-L | 0.991 | 0.411 |
| Controlvs. EA-H | <0.001 | 2.290 |
| Control vs. EA-M | <0.001 | 2.690 |
| Control vs. EA-L | 0.150 | 1.212 |
| Control vs. BU-H | <0.001 | 2.658 |
| Control vs. BU-M | <0.001 | 2.902 |
| Control vs. BU-L | <0.001 | 2.224 |
| Control vs. WA-H | <0.001 | 3.624 |
| Control vs. WA-M | <0.001 | 2.924 |
| Control vs. WA-L | 0.004 | 1.880 |

Table S4. Pairwise comparisons of IL-6 between drug-treated groups and the control group in the acetic acid-induced writhing test: *p* values, and Cohen’s *d* effect sizes

| Dunnett's multiple comparisons test | Adjusted *P* value | Cohen’s *d* |
| --- | --- | --- |
| Model vs. Control | <0.001 | 3.147 |
| Model vs. DD | <0.001 | 3.133 |
| Model vs. ET-H | <0.001 | 3.156 |
| Model vs. ET-L | <0.001 | 3.023 |
| Model vs. EA-H | <0.001 | 4.689 |
| Model vs. EA-L | <0.001 | 3.877 |
| Model vs. BU-H | <0.001 | 3.949 |
| Model vs. BU-L | <0.001 | 4.296 |
| Model vs. WA-H | <0.001 | 4.455 |
| Model vs. WA-L | <0.001 | 4.603 |
| DD vs. WA-L | <0.001 | 2.361 |

Table S5. Pairwise comparisons of COX-2 between drug-treated groups and the control group in the acetic acid-induced writhing test: *p* values, and Cohen’s *d* effect sizes

| Dunnett's multiple comparisons test | Adjusted *P* value | Cohen’s *d* |
| --- | --- | --- |
| Model vs. Control | 0.172 | 1.123 |
| Model vs. DD | 0.204 | 1.081 |
| Model vs. ET-H | <0.001 | 3.463 |
| Model vs. ET-L | 0.126 | 1.197 |
| Model vs. EA-H | <0.001 | 3.697 |
| Model vs. EA-L | <0.001 | 2.254 |
| Model vs. BU-H | 0.0511 | 1.388 |
| Model vs. BU-L | <0.001 | 2.440 |
| Model vs. WA-H | <0.001 | 2.890 |
| Model vs. WA-L | <0.001 | 2.734 |
| DD vs. WA-L | 0.012 | 1.652 |

Table S6. Pairwise comparisons of RF between drug-treated groups and the control group in the acetic acid-induced writhing test: *p* values, and Cohen’s *d* effect sizes

| Dunnett's multiple comparisons test | Adjusted *P* value | Cohen’s *d* |
| --- | --- | --- |
| Model vs. Control | 0.269 | 1.008 |
| Model vs. DD | <0.001 | 2.755 |
| Model vs. ET-H | <0.001 | 2.570 |
| Model vs. ET-L | <0.001 | 1.967 |
| Model vs. EA-H | <0.001 | 5.777 |
| Model vs. EA-L | <0.001 | 6.083 |
| Model vs. BU-H | <0.001 | 3.406 |
| Model vs. BU-L | <0.001 | 4.002 |
| Model vs. WA-H | <0.001 | 4.710 |
| Model vs. WA-L | <0.001 | 4.341 |
| DD vs. WA-L | 0.018 | 1.585 |

Table S7. Pairwise comparisons of TNF-α between drug-treated groups and the control group in the acetic acid-induced writhing test: *p* values, and Cohen’s *d* effect sizes

| Dunnett's multiple comparisons test | Adjusted *P* value | Cohen’s *d* |
| --- | --- | --- |
| Model vs. Control | <0.001 | 2.679 |
| Model vs. DD | 0.901 | 0.526 |
| Model vs. ET-H | 0.028 | 1.503 |
| Model vs. ET-L | 0.718 | 0.672 |
| Model vs. EA-H | 0.005 | 1.781 |
| Model vs. EA-L | 0.047 | 1.403 |
| Model vs. BU-H | 0.989 | 0.369 |
| Model vs. BU-L | 0.158 | 1.145 |
| Model vs. WA-H | 0.025 | 1.522 |
| Model vs. WA-L | <0.001 | 2.393 |
| DD vs. WA-L | <0.001 | 1.867 |
